# Engineering Electrode Polarity for Enhancing In Situ Generation of Hydroxyl Radicals Using Granular Activated Carbon

**DOI:** 10.3390/catal14010052

**Published:** 2024-01-11

**Authors:** Stephanie Sarrouf, Amir Taqieddin, Muhammad Fahad Ehsan, Akram N. Alshawabkeh

**Affiliations:** 1 Department of Civil & Environmental Engineering, Northeastern University, Boston, MA 02115, USA; 2 Department of Mechanical & Industrial Engineering, Northeastern University, Boston, MA 02115, USA

**Keywords:** granular activated carbon, surface modification, polarity reversal, hydrogen peroxide, hydroxyl radical

## Abstract

Recently, granular activated carbon (GAC) has shown its effectiveness as a cathode material for in situ ROS generation. Here, we present an electrochemically modified GAC cathode using electrode polarity reversal (PR) approach for enhanced H_2_O_2_ decomposition via 2-electron oxygen reduction reaction (2e-ORR). The successful GAC modification using PR necessitates tuning of the operational parameters such as frequency, current, and time intervals between the PR cycles. This modification enhances the GAC hydrophilicity by increasing the density of surface oxygen functionalities. After optimization of the electrode polarity, using the 20 (No PR)-2 (PR) interval and 140 mA current intensity, the •OH concentration reaches 38.9 μM compared to the control (No PR) (28.14 μM). Subsequently, we evaluated the enhanced •OH generation for the removal of glyphosate, a persistent pesticide used as a model contaminant. The modified GAC using PR removed 67.6% of glyphosate compared to 40.6% by the unmodified GAC without PR, respectively. The findings from this study will advance the utilization of GAC for in situ ROS synthesis, which will have direct implications on increasing the effectiveness of electrochemical water treatment systems.

## 1. Introduction

The inevitable intensification of industrial activities has led to severe environmental pollution consequences in water, soil, and air. Over the years, the accumulation of different recalcitrant pollutants in conventional biological and chemical treatments has directed the research community to further the research on electrochemical advanced oxidation processes (EAOPs) [1]. Significant efforts focused on the mechanisms of electrochemical reactive species production, its application for the removal of aqueous organic pollutants, and its optimization [2–4]. Electrochemical water treatment methods rely on the generation and employment of powerful oxidants, such as hydrogen peroxide (H_2_O_2_) and hydroxyl radicals (•OH), to degrade contaminants in water treatment [3,4]. The efficient generation of these oxidizing agents depends primarily on the cathodic material and its surface chemistry [5–8].

Over the past decade, several materials have been designed, optimized, and used toward achieving efficient generation of H_2_O_2_ and •OH for water treatment [9–12]. Carbonaceous materials are considered one of the promising cathodic candidates because of their stability, non-toxicity, good conductivity, cost-effectiveness, and chemical resistance characteristics [3,13–16]. Given their advantages, several carbonaceous materials have been used in the electro-Fenton (EF) processes for water treatment such as graphite [17], carbon felt [18], activated carbon (AC) [19], and carbon-polytetrafluoroethylene (PTFE) [20]. AC has been used widely for the removal of pollutants from water [21–24], because of its chemical adsorption, exceptional high surface area, wide range of surface group, and regeneration characteristics [21,25,26]. Despite its several advantages, AC can exhibit poor kinetics such as activity, selectivity, and stability towards the 2-electron oxygen reduction reaction (2e-ORR) [27,28], which affects H_2_O_2_ production; hence, the generation of •OH.

To overcome these limitations, a significant number of modifications have been conducted to improve the surface chemistry of ACs towards enhancing their electrocatalytic activity [29–32]. Given that the generation of H_2_O_2_ and •OH occurs via interfacial electron transfer at the surface of the electrode, engineering and manipulating the surface chemistry of the AC electrodes are key routes to effectively enhance the electrochemical processes. Surface chemical properties of the electrodes such as their wetting and adsorption are highly dependent on their content of the chemisorbed oxygen at the surface which can be found in the form of various surface functional groups. Different types of modifications are generally used to enhance the concentration and nature of the active sites on the surface. The first modification approach is based on the impregnation of the AC with catalysts such as acetylene black [33], carbon nanotubes [11], or metal oxides (i.e., MnO_2_ [34], IrO_2_ [35], RuO_2_ [36]). The modified carbon-based catalysts are able to control the cleavage of the O-O bonds by altering the chemisorption characteristics of O_2_ [37]. The catalytically modified AC then acquires a high activity and selectivity in the 2e-ORR for H_2_O_2_ production [37]. Alternatively, heteroatom-doping on the surface of the electrode using O [38], N [39], or F [40] produces impurity defects to improve ORR performance, but the catalytic active center exhibits controversy [37]. Introducing surface oxygen groups (OGs) at the electrode surface can also be achieved by either introducing strong oxidants [41] (H_2_O_2_, AgNO_3_, H_2_PtCl_6_, HNO_3_) or electrochemical oxidation [4]. The introduction of OGs is an efficient and facile way to increase the electrical conductivity and electrocatalytic activity of the electrode due to the enhancement of wettability [42,43]. The modification of ACs using strong oxidants can be difficult to control for water treatment due to the limitations of operating both pH and temperature [44]. Introducing OGs via electrochemical oxidation has several advantages [45]: (i) one of the reagents is the electron, which can be provided by a direct current (DC) source; (ii) the treatment can be easily applied and regulated; and (iii) redox processes are selective and can be controlled easily by the electrode potential. Although a significant amount of research has been conducted to introduce OGs on carbonaceous surfaces by electrooxidation [8,44,46], few studies focused on enhancing the surface groups of granular activated carbon (GAC) to produce H_2_O_2_ and •OH. Lately, GAC has been used widely as an effective way for water treatment due to its adsorption characteristic [47,48]. One of the issues of utilizing GAC is its poor absorption and selectivity when it comes to certain contaminants [49]. To overcome this limitation, the recent trend is focused on different treatment methods (e.g., electrochemical oxidation) that involve physicochemical modifications of the GAC properties using strong acids as electrochemical oxidizing agents [29,50]. In these few studies, strong acids were mainly used as electrochemical oxidizing agents during the surface modification of GAC, which makes these processes difficult to control, costly, and hard to implement on large scales.

Recently, polarity reversal (PR) has received considerable attention to achieve sequential cathode modification and H_2_O_2_ generation [4]. Electrode PR is defined as an approach to alternate the anode and cathode polarity at different time intervals [51]. This technique has been previously used as a practical and controllable approach in the electrokinetic remediation applications of heterogeneous media [52]. In a recent study [4], polarity reversal resulted in an enhanced H_2_O_2_ generation using the graphite felt cathode, consequently improving the degradation of reactive blue 19 (RB19) and ibuprofen (IBP). Recently, Ansari et al. [53] performed PR along with surface activation using acid treatment on a cathode made primarily of banana peel-derived biochar to enhance the concentration of oxygen surface groups, hence improving the generation of H_2_O_2_. While PR technique has been used for improving the modification of electrodes in the presence of acidic oxidants, it has not been evaluated for GAC electrode enhancement, and the production of H_2_O_2_ and •OH in an Fe-free EF process operating at a neutral pH. This method could be efficient for tuning the wettability of the carbonaceous surface, hence obtaining an optimal catalytic performance of GAC electrodes.

Thus, this paper provides a systematic investigation of achieving electrochemical modification of AC-PTFE via PR. This technique can be applied in situ for enhancing the decomposition of H_2_O_2_ into •OH in an acid-free solution. This study analyzes the influence of the electrode polarity (anodic or cathodic) and the effect of specific parameters of the polarity reversal such as frequency, duration of intervals, and the intensity of the applied current in an Fe-free EF process for the generation of •OH. This study would provide a way to implement polarity reversal on carbonaceous surfaces to tune its wettability degree and active sites, hence increasing its effectiveness for H_2_O_2_ activation into •OH.

## 2. Results and Discussion

### 2.1. Electrogeneration of H_2_O_2_ and •OH by PR

Electrogeneration of both H_2_O_2_ and •OH were controlled by manipulating the PR frequency and the current. First, the O_2_ was generated in situ at the Ti/MMO anode through the O_2_ evolution reactions as follows:

(1) $$2{\text{H}}_{2}\text{O}\to {\text{O}}_{2}+4{\text{e}}^{-}+4{\text{H}}^{+}\text{(anodic surface) }.$$

The produced O_2_ becomes reduced via 2e-ORR at the cathodic surface to generate H_2_O_2_ as follows:

(2) $${\text{O}}_{2}+2{\text{H}}^{+}+2{\text{e}}^{-}\to {\text{H}}_{2}{\text{O}}_{2}\text{ (cathodic surface). }$$

Subsequently, the H_2_O_2_ decomposes at the cathodic surface and within the pore structure to form •OH as described in the following reaction:

(3) $${\text{H}}_{2}{\text{O}}_{2}+\text{GAC}\to \;•\text{OH}+{\text{OH}}^{-}+{\text{GAC}}^{+}(\text{cathodic surface })$$

(4) $${\text{GAC}}^{+}+{\text{H}}_{2}{\text{O}}_{2}\to \text{GAC}+{\text{H}}^{+}+{\text{HO}}_{2}•(\text{ cathodic surface })$$

where GAC^+^ is the oxidized form of GAC. Equation (3) represents the formation of •OH in an electron-Fenton-like process (i.e., iron-free). The •OH concentration was measured through the concentration of 4-HBA as follows:

(5) [•OH]=5.87[4−HBA]

For investigating the PR effect on the generation of H_2_O_2_ and •OH, we measured their concentrations under various testing conditions. Table 1 summarizes the testing variables used during the various experiments. The obtained concentrations were measured using spectrophotometer and HPLC for H_2_O_2_ and •OH, respectively (see Section 3 for more details). The PR frequency (cycles/h) is calculated as follows:

(6) $$\text{ PR frequency }=\frac{\text{ Number of cycles }}{\left(t_{\text{o}}+t_{\text{r}}\right)}$$

where *t*_*o*_ is the duration of the original electrode polarity (h) and *t*_*r*_ is the duration of the reversed electrode polarity (h). The following subsections present the obtained results of H_2_O_2_ and •OH generation via the above reactions.

The current efficiency (*CE*) is calculated as follows:

(7) $$\text{CE\%}=\frac{{\text{nFc}}_{\text{i}}\text{V}}{\text{It}}100\text{\%}$$

where *n* is the number of transferred electrons which is set to 2 (Equation (2)), *F* (96,486 C/mol) is the Faraday’s constant, *c*_*i*_ (mol/L) is the concentration of the chemical species where *i* is either H_2_O_2_ or •OH, *I* (A) is the externally applied current, *V* is the liquid volume in the reactor (L), and *t* is the reaction time (s).

#### 2.1.1. Influence of PR Modification

**Figure 1.**
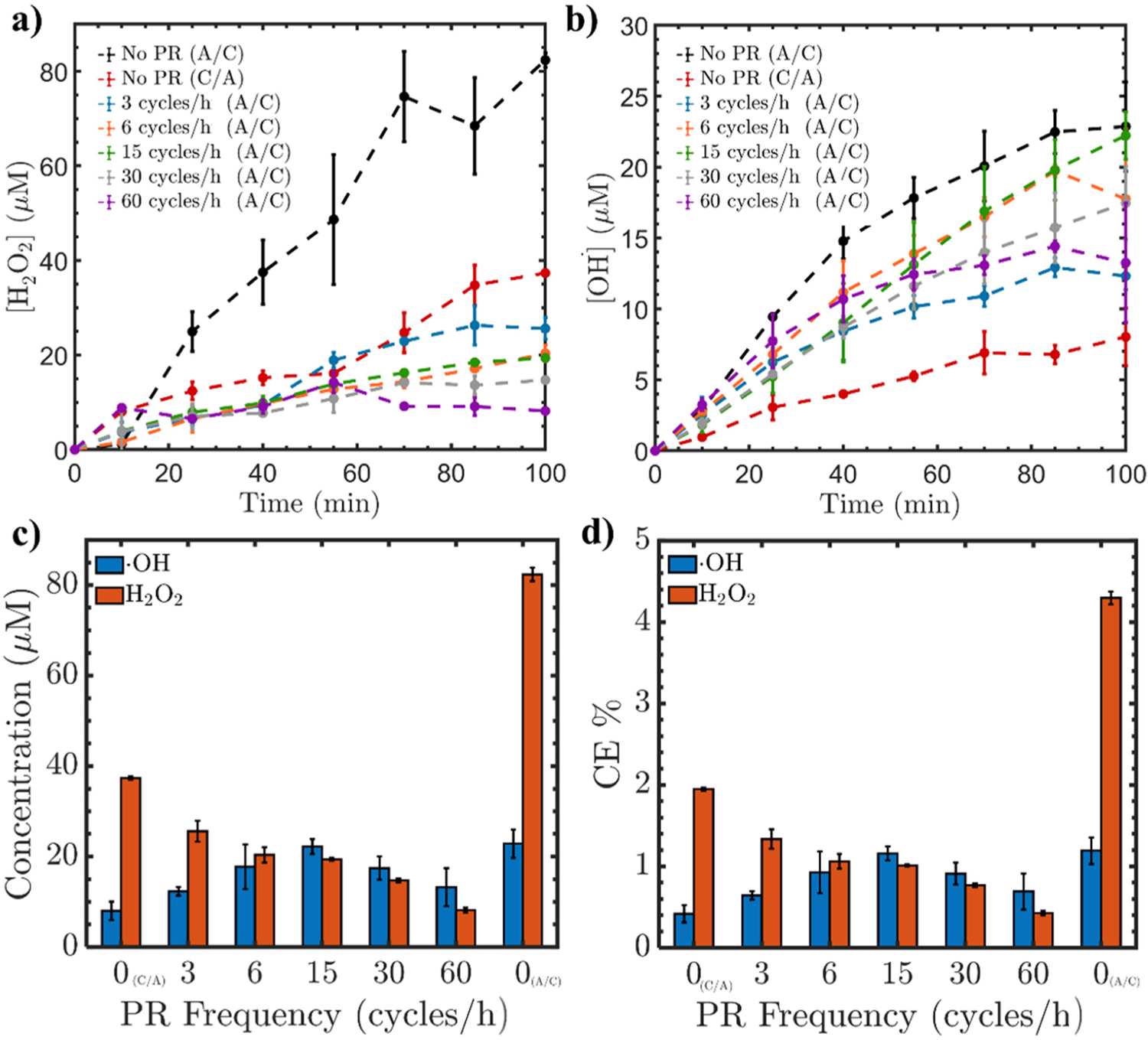
The experimentally measured concentrations of (**a**) H_2_O_2_ and (**b**) •OH as a function of time, along with (**c**) the final concentrations of H_2_O_2_ and •OH, and (**d**) CE after 100 min under varying PR frequencies using a current intensity of 60 mA.

In general, the H_2_O_2_ concentration profiles obtained over time during the application of PR exhibit lower concentration values compared to the configuration without PR (i.e., A/C or C/A). The measured concentration of H_2_O_2_ after 130 min (see Figure 1a) exhibits the highest value when no PR is used in the case of A/C arrangement while the lowest value is observed when the PR frequency is 60 cycles/h (A/C). The H_2_O_2_ concentration between 60 and 100 min depicts an inverse relation to the PR frequency (e.g., as the PR frequency increases, the H_2_O_2_ concentration decreases at a given time). Specifically, Figure 1c and 1d show, respectively, the obtained concentration and CE of both H_2_O_2_ and •OH at time of 100 min using the various PR frequency testing conditions. The maximum obtained CE is about 4% which is in agreement with the previous studies [54]. Clearly, the concentration of H_2_O_2_ decreases as the PR frequency increases. This can be explained by the insufficient reaction time for the electrode to act as either the anode or cathode. In other words, when the PR frequency is high, it means that the electrode will have a shorter time to act as the anode/cathode and vice versa. This short time limits the reaction of H_2_O_2_ generation and OG formation at the surface of the GAC, leading to lower concentrations of H_2_O_2_ as the PR frequency increases [8].

Figure 1b represents the •OH concentration as a function of time under different PR frequencies. While the H_2_O_2_ concentration showed an inverse relationship with the PR frequency, the obtained concentrations of •OH showed no direct proportional relation to the PR frequency. First, the concentration of •OH exhibits a maximum value in the case of no PR testing condition with the A/C arrangement, and a minimum concentration value for the no PR testing condition with the C/A arrangement. Second, the concentration of •OH at the final time (i.e., 100 min) increases as the PR frequency increases, until it reaches the maximum at a PR frequency of 15 cycles/h, and then it starts decreasing as the PR frequency further increases. This parabola-like behavior can be related to the time duration for the GAC and Ti/MMO to serve as the anode and cathode, respectively. Oxygen evolution reaction (OER) is a four-electron process that occurs at the anode, possessing a higher kinetic barrier than the hydrogen evolution reaction [55]. The OER requires stable and active electrocatalyst materials due to the high overpotential required for the oxidation of water [56]. While GAC is considered an effective cathode, it behaves as a “nonactive” anode and does not possess an elevated overpotential for the OER [57,58]. According to Yuan et al. [59], Ti/MMO can be used as a cathode for O_2_ reduction in electro-Fenton processes. Unlike activated carbon, Ti/MMO is unable to activate H_2_O_2_ into •OH in an iron-free system [59]. Also, when increasing the PR frequency, the polarity duration and time for reactions decrease. For the 30 and the 60 cycles/h, the ratio of polarities was 2:1, respectively. The amount of charge evolved for reduction and oxidation in one cycle is dictated by the current interval. Thus, it can be assumed that 30 and 60 cycles/h are not sufficient to favor the reactions on the electrodes. Consequently, with an increase in the PR frequency to 30 and 60 cycles/h, there is a decrease in the •OH concentration. This can be ascribed to the limited capability of Ti/MMO to effectively generate anodic O_2_, resulting in a lower concentration of H_2_O_2_ that is insufficient for •OH activation.

#### 2.1.2. Influence of Current Intensity

**Figure 2.**
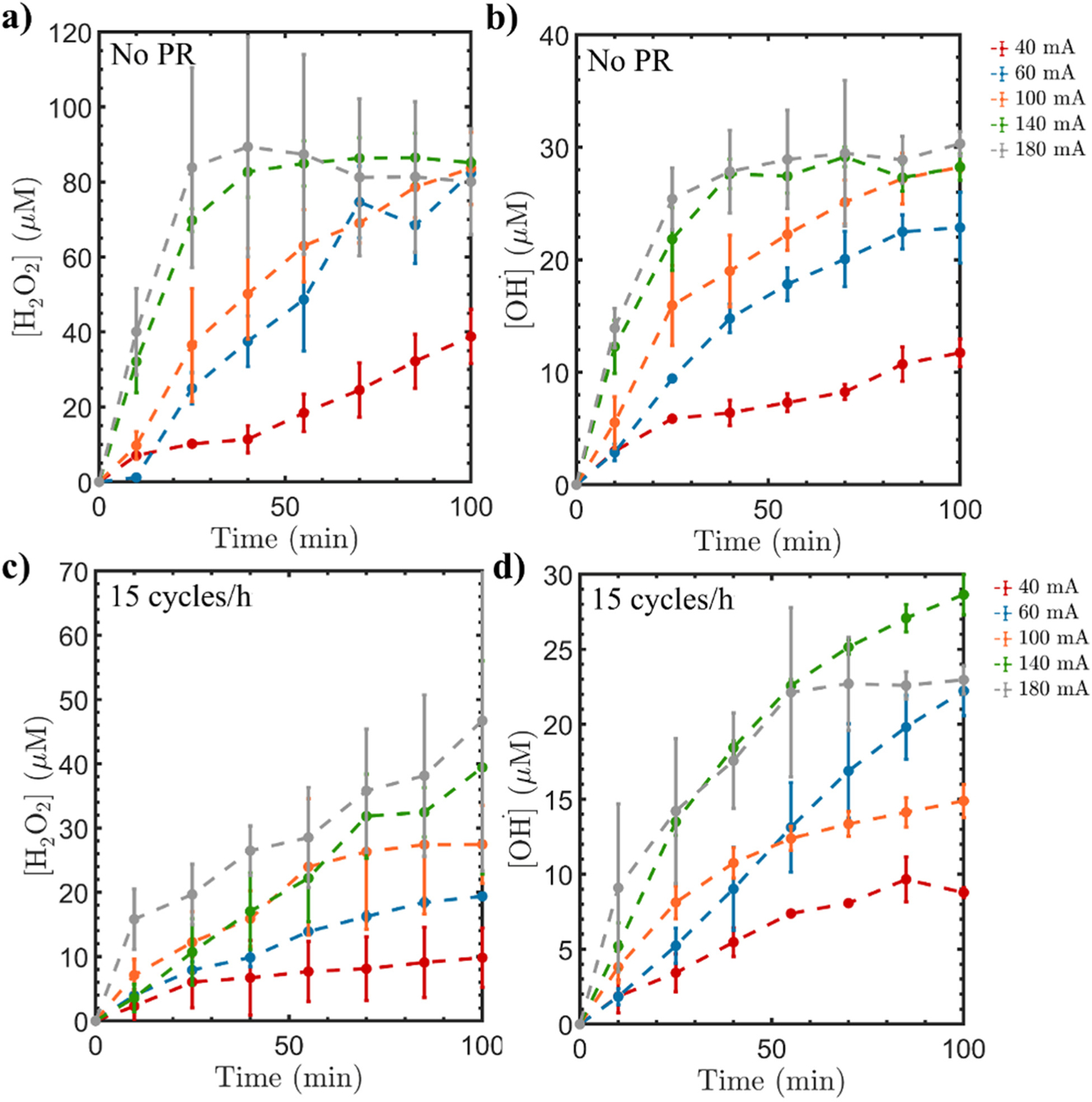
(**a**,**b**) Variation of H_2_O_2_ and •OH concentration with no PR modification under the influence of different current intensities, and (**c**,**d**) influence of PR using 15 cycles/h as a modification on H_2_O_2_ and •OH production under different current intensities.

Figure 2a,b exhibit the generation of H_2_O_2_ and •OH, respectively, without applying PR conditions. The experiments were conducted under different current intensities as listed in Table 1. It can be noticed from both figures that the concentrations of H_2_O_2_ and •OH increase as the current increases from 40 mA to 180 mA. This is expected and can be related to Faraday’s law where the amount of the generated H_2_O_2_ and •OH, *m*, can be explained by the following:

(8) $$\text{m}=\frac{\text{I}\times \text{t}\times M_{\text{w}}}{\text{n}\times \text{F}}$$

where I is the electrical current, t indicates the reaction time, M_w_ is the molar mass of the H_2_O_2_ or •OH, n is the number of electrons transferred, and F is the Faraday’s constant. This indicates that as the time progresses, the amounts of H_2_O_2_ and •OH increase with increasing current, hence achieving the highest concentrations at 180 mA.

Next, we applied 15 cycles/h PR on the electrochemical systems with varying current intensities to compare the obtained results with no PR. The PR frequency of 15 cycles/h was selected among the previous cycles (3, 6, 30, and 60 cycles/h) since it showed the best performance toward •OH generation. Figure 2c,d show the concentrations of H_2_O_2_ and •OH, respectively, for the various current intensities under the influence of PR. Similar to the no PR configuration, the H_2_O_2_ concentration increases as the current intensity increases. However, the obtained concentration profiles as a function of time have lower values when the PR is applied compared to the cases without PR, as previously discussed in Section 2.1.1. Specifically, the concentration of H_2_O_2_ under 15 cycles/h PR modification is notably lower compared to the H_2_O_2_ concentration generated by the cathode without PR under 180 mA. This can be attributed to the increase in the current, which leads to a gradual increase in the degree of oxidation but to a lesser extent when PR is applied. Additionally, it might result in the anodic oxidation of H_2_O_2_ to generate H_2_O [3,44]. Higher current can also mineralize the GAC surface OGs, hence limiting the H_2_O_2_ and •OH production [4]. On the contrary, establishing a direct relationship between the •OH concentration and the current intensity is challenging. The •OH generation exhibits distinct trends during PR experiments. Specifically, at 40 mA, it is the lowest, followed by 100 mA. Surprisingly, the •OH concentration at 60 mA surpasses that at 100 mA. This indicates a non-linear relationship between •OH concentrations and current intensity. This trend persists even at higher current intensities (140 and 180 mA) where the •OH concentrations remain nearly similar during the initial 50 min of the experiment. However, the •OH concentrations at 140 mA exceed that at 180 mA after the initial 50 min duration.

Since the Ti/MMO is an active anode for OER, higher current intensities facilitate the formation of oxides on the surface, thereby aiding the OER [60]. Hence, the complex correlation between the •OH concentration and the current intensities in the presence of PR can be explained by the accumulation of O_2_ bubbles at the cathode surface of GAC-PTFE, which can hinder the electrode’s surface activity [61]. At higher current intensities, the kinetic rate of O_2_ production is faster, the gas bubbles will tend to accumulate faster underneath the GAC, resulting in electrowetting which hinders the fresh electrolyte penetrate GAC (see Figure S3). Furthermore, frequent alteration of electrode polarity (15 cycles/h, with 4 min as cathode and 4 min as the anode) indicates that the Ti/MMO electrode is not capable of generating O_2_ in a continuous manner. This will affect the rate of bubble growth at the electrode surface since it depends on the concentration ratio of O_2_ between the electrolyte and the bubble [62]. If the growth rate is not fast enough, then the bubble will reside for a longer time on the electrode surface given that it needs more time to reach sufficient size before leaving the electrode [62]. However, when the Ti/MMO is a constant anode, the oxygen will be continuously generated at the Ti/MMO anode and as the bubble continues to grow larger, the probability of it detaching from the electrode surface increases, allowing fresh electrolytes to interact with the cathode and produce more •OH. It also should be noted that the bubble size should not be very large so it can pass through the mesopores of GAC. Otherwise, if the bubble does not pass through the electrode, it will reside for a long time and its size will be become very large due to the coalescence of bubbles and this will lead to large coverage of the electrode surface, reducing its electroactive area [63].

In addition to the bubble effects on the electrochemical reactions for •OH production, competing reactions and their associated potential related to different current intensities can nonlinearly influence the •OH production. Typically, the current response towards ORR can increase when the current applied is higher [4]. However, parasitic reactions also evolve as the current increases. The possible competing reactions at the GAC cathode can be summarized as follows:

(9) $$2{\text{H}}_{2}{\text{O}}_{2}\to {\text{O}}_{2}(\text{g})+2{\text{H}}_{2}\text{O}$$

(10) $${\text{H}}_{2}{\text{O}}_{2}+2{\text{H}}^{+}+2{\text{e}}^{-}\to 2{\text{H}}_{2}\text{O}$$

(11a) $${\text{H}}_{2}{\text{O}}_{2}\to {\text{HO}}_{2}•+{\text{H}}^{+}+{\text{e}}^{-}$$

(11b) $${\text{HO}}_{2}•\to {\text{O}}_{2}(\text{g})+{\text{H}}^{+}+{\text{e}}^{-}$$

These reactions indicate the several pathways that H_2_O_2_ can follow during its decomposition, which include disproportion (Equation (9)), cathodic reduction (Equation (10)), and anodic oxidation (Equation (11)) [4]. Moreover, the more the H_2_O_2_ decomposes, the harder it becomes for the generation to take place. Therefore, in the following section, the reaction time of the anode/cathode and cathode/anode is tuned to decrease the occurrence of the competing reactions (Equations (9)–(11)).

#### 2.1.3. Influence of Reaction Time

**Figure 3.**
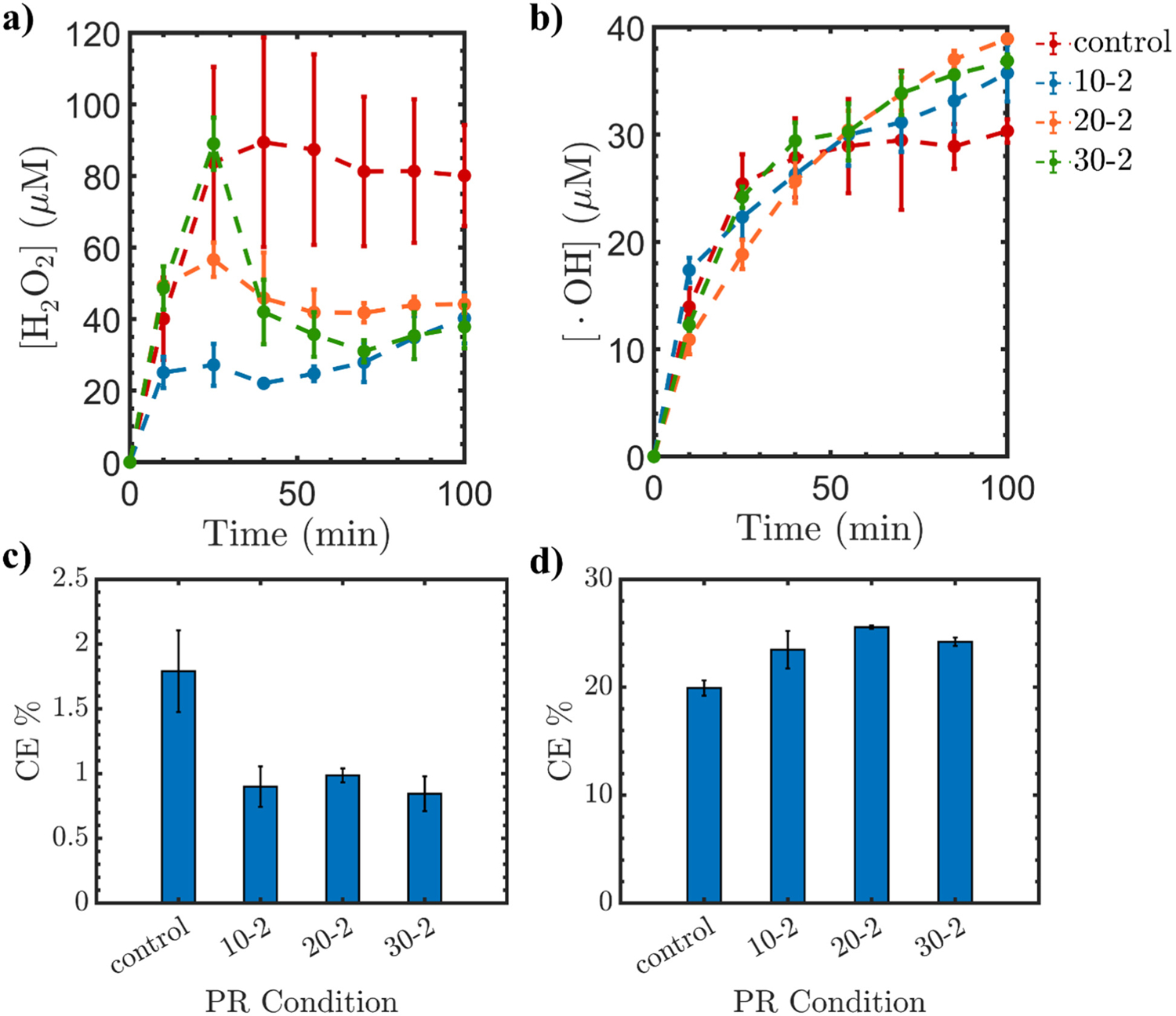
The obtained (**a**,**b**) concentration and (**c**,**d**) CE for H_2_O_2_ and •OH, respectively, with different PR intervals duration under a current intensity of 140 mA for the intervals of 10–2, 20–2, and 20–3. The first number corresponds to the time (min) of no PR condition and the second number corresponds to the time (min) of PR interval.

Figure 3 presents the obtained results where the time of the PR was tuned. We varied the polarity interval as summarized in Table 1. All the experiments were performed under a current of 140 mA since it produced the highest concentration of •OH under PR application as discussed in the previous section. The obtained results show that the control condition (i.e., no PR) produces the highest concentration of H_2_O_2_ as a function of time. The time interval has two indices: the first one corresponds to the interval of having no PR (i.e., GAC is assigned as the cathode) and the second index corresponds to the interval of applying PR (i.e., GAC can be either the cathode or anode). The obtained results show that the H_2_O_2_ increases as a function of time with a rate similar to the no PR trend until the PR testing condition is turned on. Specifically, the trend for the case of 10–2 (i.e., 10 min no PR and 2 min PR) conveys that the H_2_O_2_ concentration increases during 10 min without PR and then it starts to decrease.

Upon increasing the cathodic time for the GAC from 10 to 20 and then to 30 min, the concentration of H_2_O_2_ increases gradually, achieving a higher concentration of ~89 μM. When the no PR condition is prolonged (i.e., 30–2), it extends the period during which GAC acts as the cathode, leading to increased H_2_O_2_ production. Conversely, with the introduction of PR, the H_2_O_2_ concentration decreases since ORR over Ti/MMO is kinetically not feasible. Instead, the Ti/MMO, acting as a cathode, facilitates the hydrogen evolution reaction (HER) (Equation (12)) instead of the oxygen evolution reaction (OER) [64]. However, the concentration of •OH keeps increasing even when the PR is on due to the H_2_O_2_ high lifetime [65]. During no PR application, the generated H_2_O_2_ remains adsorbed on the surface of the GAC until it becomes decomposed into •OH. The conversion of H_2_O_2_ into •OH is a surface phenomenon that is facilitated by the microelectrode GAC particles and is not governed by the electrode polarity. Therefore, it is the adsorbed H_2_O_2_ during PR that keeps decomposing into •OH.


(12) $$2{\text{H}}_{2}\text{O}\to {\text{H}}_{2}(\text{g})+2{\text{OH}}^{-}$$

**Figure 4.**
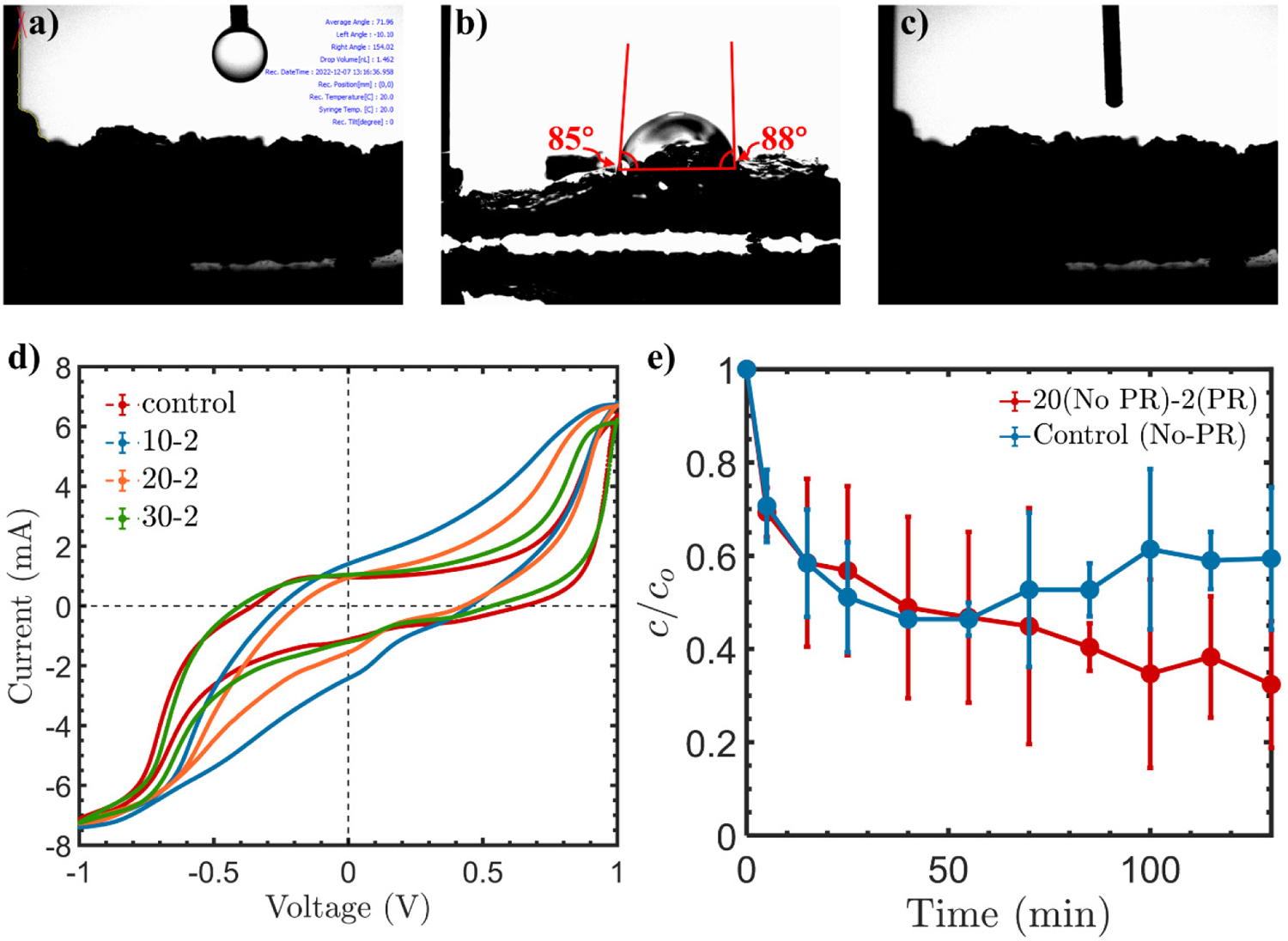
(**a**–**c**) Contact angle measurement showing the drop of water, on the surface of the control, and going through the 20 min (No PR)-2 min (PR) surface; (**d**) CV curves recorded in the scan range of −1 to 1 V and (**e**) Glyphosate degradation under control (No PR) and 20 min (No PR)-2 min (PR).

After the PR is off, the freshly produced H_2_O_2_ is effectively decomposed into •OH due to the restoration of surface OGs on the GAC after the PR is applied, since anodic current increases the amount of OGs [29]. The increase in active surface sites, mainly through OGs, will efficiently increase the •OH generation through H_2_O_2_ decomposition by eliminating the competing reactions (see Equations (9)–(11)) [23,66–68]. In addition, the high concentration of H_2_O_2_ formed will serve as an oxidizing chemical when PR takes place to introduce the surface OGs, including carboxyl, hydroxyl, carbonyl, and lactone functionalities [69]. Figure 4b reveals that the obtained •OH concentrations are higher for the PR testing conditions compared to the control case. The concentrations of •OH at intervals 20 min (No PR)-2 min (PR) and 30 min (No PR)-2 min (PR) increase with time to reach a value around 38.9 and 36.8 μM, respectively, compared to 35.7 μM under 10–2 PR and 28.14 μM under control. A 38.2% increase in the generation of •OH when using 20 min (No PR)-2 min (PR) was achieved.

The obtained results show that the CE value of H_2_O_2_ for the control case (No PR) is higher than the cases of different PR intervals. However, the CE values of •OH are higher for the PR cases compared to the control system. The increase in the OGs enhances the catalytic activity of the GAC. Therefore, providing the adequate time for the cathodic and anodic reactions on the GAC is vital for regenerating its surface functionalities via in situ modification toward efficient H_2_O_2_ activation [4].

This innovative approach assures continuous radical generation and reduces the loss of catalytic activity for water treatment applications.

### 2.2. Characterization: Contact Angle Measurement and Cyclic Voltammetry

The introduction of OGs onto carbon surfaces increases its electrocatalytic activity due to the formation of a hydrophilic surface [70]. To test the hydrophilicity of the surface, contact angle measurements were performed on the controlled GAC electrode (No PR) and the modified (i.e., 20–2) GAC electrode surface that were previously used in Figure 3. Figure 4a demonstrates the droplet of water at the initial stage before contact with the GAC surface. Figure 4b displays the droplet of water resting at the GAC electrode (No PR) surface at an average angle of 86°. Since the water droplet rested on the surface at an angle < 90°, this indicates that the GAC surface exhausted part of its OGs, which hinders its surface activity due to reduced surface hydrophilicity [9]. Figure 4c demonstrates the surface of the GAC electrode with 20–2 (20 min no PR and 2 min PR). The water was absorbed by the GAC surface instantly, which made capturing the water droplet hard. Since the water droplet rested at an angle of 86° on the GAC surface (No PR) and was immediately absorbed by the surface of the GAC (20 min no PR and 2 min PR), this indicates an increase in the surface’s hydrophilicity after performing PR at a certain time interval. The increase in hydrophilicity allows enhanced mass transfer of O_2_ to the electrode while preventing accumulation of O_2_ bubbles leading to electrowetting [70].

Figure 4d shows the control, 10–2, 20–2, and 30–2 CV curves. The control curve exhibits symmetry in the positive and negative direction and no hysteresis. The 10–2, 20–2, and 30–2 modifications do not present similarities in both directions; they are different in the positive direction. This implicates a change in the redox reactions taking place at the GAC’s surface. Looking at the voltage between 0 and 0.5 V, the control (No PR) has the lowest current. Also, looking in the negative direction from 0 to −0.5 V, the control (No PR) exhibits the smallest current values. From the other end, the 10–2, 20–2, and 30–2 exhibited a higher current response in both directions than the control (No PR). Compared with Zhou et al. [70], the carbon with lower hydrophilic surface area exhibited a lower current response than the one with more hydrophilic area. Based on this result, it can be concluded that the control (No PR) exhibits less hydrophilic characteristics that hamper O_2_ mass transfer, hence hindering the effective H_2_O_2_ formation and its subsequent decomposition into •OH. This suggests the significance of precisely engineering the electrode polarity, because the results indicate that the efficiency of the PR is dependent on the time of each polarity. The adequate application of PR can enhance the wettability of the GAC’s surface; hence, the effective decomposition of H_2_O_2_ into •OH can take place and hinders the occurrence of competing reactions.

After optimizing the PR for enhanced •OH generation, glyphosate (a commonly used herbicide) was used as the model contaminant to test the efficiency of the modified GAC toward the degradation of glyphosate in an electrochemical flow through a reaction under a current of 140 mA. The removal of glyphosate was evaluated for the control (No PR) and the 20–2 GAC, respectively. Figure 4d demonstrates that when the PR condition of 20–2 is applied, the removal of the glyphosate increases from 40.6% to 67.6% in the span of 130 min. The removal of glyphosate is almost similar in both cases up to 45 mins. Beyond this point, the glyphosate removal under the control condition (No PR) starts approaching a constant removal percentage. In contrast, the GAC subjected to the PR treatment of 20–2 provides a continuous removal of the glyphosate as the reaction proceeds. This indicates that the PR with optimized parameters is able to significantly enhance the contaminant removal [71,72]. This is consistent with the results presented in Figure 4b, where the higher production of •OH was obtained when 20–2 condition was used, and the removal rate exhibited a similar behavior to the production of hydroxyl radicals. In comparison to other handful of studies that were carried out for the EF regeneration of GAC, several efficiency metrics were used such as the removal of contaminants and current efficiency. In some of the studies reported, optimal removal of orange II could reach 66% when using GAC that was modified with 0.9 mM Fe^2+^ [73]. In another study performed by Trellu et al., the EF regeneration of phenol-saturated activated carbon reached 70% removal [74]. In a study performed by Li et al., where GAC, as a heterogeneous catalyst, only achieved 25.4% of acid orange 7 removal [75]. Finally, in a study performed by Mines et al., where a nanoporous polymetric network was grafted to activated carbon granules for the removal of nitrobenzene, a 63.6% total removal was achieved [76].

## 3. Methodology

All chemicals used in this study were of analytical grade and purchased from Fisher Scientific, Waltham, MA, USA which include the following: sodium sulfate (Na_2_SO_4_ anhydrous, ≥99%), calcium sulfate (CaSO_4_ anhydrous, 99.9%), titanium sulfate (Ti(SO_4_)_2_, 99.9%), hydrogen peroxide (H_2_O_2_, 30% solution), reagent alcohol (90%, HPLC grade), water (HPLC grade), methanol (HPLC grade), phosphoric acid (H_3_PO_4_), sulfuric acid (H_2_SO_4_), and benzoic acid (C_7_H_6_O_2_). PTFE was purchased from Fuel Cell Earth, Woburn, MA, USA. Deionized (DI) water (18.2 MW cm) was obtained from a Millipore Milli-Q system and was used in all the experiments. Ti/MMO (Ti/IrO_2_-Ta_2_O_5_-3N international) and granular activated carbon (−20 + 40 mesh, Alfa Aesar, Ward Hill, MA, USA) were used as electrodes. The Ti/MMO electrode consists of an IrO_2_ and Ta_2_O_5_ coating on a titanium mesh with a diameter of 4.3 cm.

### 3.1. Preparation of GAC Cathode

The GAC was rinsed repeatedly with DI water and dried in an oven at 80 °C for 24 h. To fabricate the GAC electrode, a ratio of 1:2:4 of GAC, PTFE, and reagent alcohol was mixed for 10 min in an ultrasonic bath to prepare a well-mixed thick paste. Alcohol was added to wet the GAC surface. The mixture was then spread onto the SS mesh and placed in the oven for annealing at 350 °C for 1 h.

### 3.2. Electrochemical Reactor

**Figure 5.**
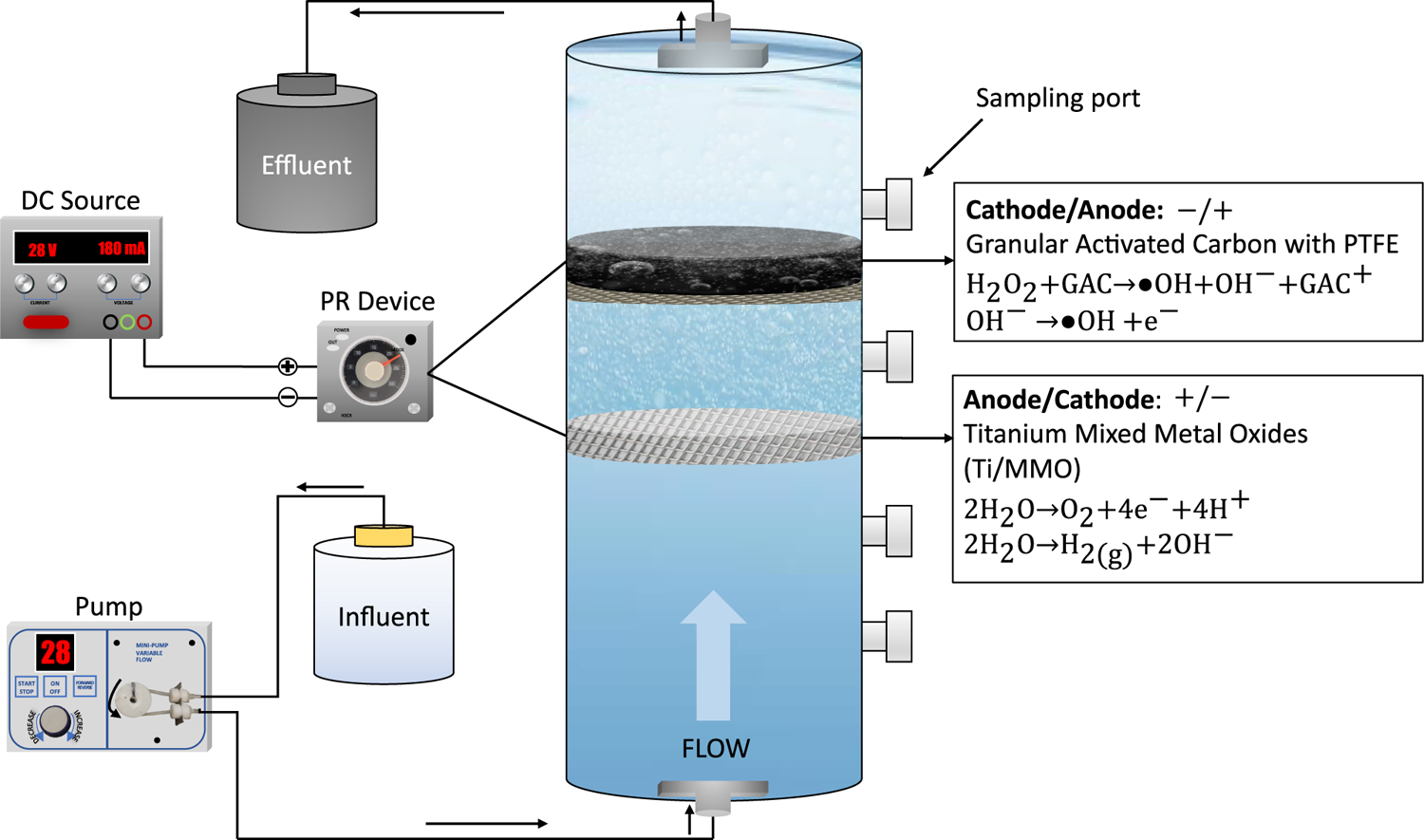
Experimental setup of the electrochemical oxidation of GAC.

The experimental setup of the electrochemical flow-through reactor with the PR device is shown in Figure 5 Once the reactor was filled, flow-through experiments were carried out. The reactor has a cylindrical column shape made of acrylic material with a height and diameter of 16 cm and 4.4 cm, respectively. Sampling ports were set at a spacing of 3 cm. The spacing between the anode (i.e., Ti/MMO) and the cathode (i.e., GAC-PTFE) was 2.5 cm. The PR frequency was set by double pole double throw (i.e., DPDT) relay made by Omron, Kyoto, Japan and its model number is H3CR-F8–300AC-100/240. The original electrode polarity is defined by using the Ti/MMO as the anode and the SS + GAC as the cathode. The abbreviation of the original electrode polarity is the anode/cathode (A/C) arrangement, and the reversed polarity is a C/A arrangement. Artificial groundwater consisting of 0.5 mM CaSO_4_ and 3 mM Na_2_SO_4_ was prepared in DI water and used as the electrolyte in all experiments. The experiments were performed at room temperature and a pH = 3.

### 3.3. Characterization of the GAC-PTFE

We initially investigated the effect of applying PR on the generation rate of H_2_O_2_ and •OH. We varied the current intensity and studied its effect on the electrode with and without PR application. We explored the effect of electrode polarity duration to optimize the electrochemical generation of •OH. The wettability of electrodes is typically characterized by the contact angle measurement of a water droplet on the surface, which is defined as the angle between the chord and the circular arc [6]. The contact angle was measured by a contact angle meter (OCA15, Dataphysics, Riverside, CA, USA). To further evaluate the GAC electrocatalytic activity towards ORR before and after PR, CV was performed in a three-electrode system using Ag/AgCl as a reference cell.

### 3.4. Analytical Methods

To quantify the concentration of H_2_O_2_, 3 mL of solution samples were collected from the port of the electrochemical reactor, and then 0.5 mL of 15 mM Ti(SO_4_)_2_ was used during the spectrophotometric analysis. Ti(SO_4_)_2_ solution was prepared by adding 1.8 g of Ti(SO_4_)_2_ in 450 mL of DI and 50 mL of H_2_SO_4_ solution and then stirred for 3 h. The concentration was then measured using the Shimadzu UV-1800 UV Spectrophotometer (Kyoto, Japan), at a wavelength of 405 nm [77]. The pH was measured by using a pH meter. Benzoic acid (BA) with a high second-order rate constant with •OH (4.2 × 10^−9^M^−1s−1^) was used for the semi-quantitative measurement of •OH [4]. For the quantification of both BA and hydroxylated isomer byproducts, a high-performance liquid chromatography with a UV detector was used. The wavelength at which the 4-hydroxybenzoic acid was detected is 254 nm using an Agilent 1260 Infinity Quaternary LC, Santa Clara, CA, USA with an eluent of 80% HPLC grade water and 20% methanol adjusted with phosphoric acid to reach a pH of 2.3.

## 4. Conclusions

Engineered PR frequencies is a green technique for enhancing the surface functionalities of GAC and promoting an efficient decomposition of H_2_O_2_ into •OH. The tuning of the OGs of the GAC’s surface through PR frequencies and current intensity allowed for a higher concentration of •OH that led to an enhanced wettability and faster oxidation of glyphosate. The enhanced adsorption by the GAC allowed for better contact between the electrolyte and the active sites of the GAC as well. Consequently, tuning the PR method can make the GAC acquire a larger surface of active sites through the form of additional OGs and workability of these active sites without exceeding the limit of their oxidation through an adequate current intensity. Hence, the selectivity of the GAC increases towards the 2e-ORR, as well as the efficient decomposition of H_2_O_2_ to •OH to degrade contaminants more efficiently.

## Supplementary Material

SI

## Figures and Tables

**Table 1.** Summary of the testing variables used to investigate the H_2_O_2_ and •OH generation.

Variable	Unit	Values	Conditions
PR frequency	Cycles/h	3; 6; 15; 30; 60	10 mM BA; 60 mA
Current intensity	mA	40; 60; 100; 140; 180	10 mM BA; No PR; 15 cycles/h
PR	-	A/C; C/A	10 mM BA; 60 mA
Time interval	mins	10–2; 20–2; 30–2	10 mM BA; 140 mA

## Data Availability

The generated data are available from the corresponding author upon request.
