# Supplementary material for: Engineering Electrode Polarity for Enhancing In Situ Generation of Hydroxyl Radicals Using Granular Activated Carbon
Source: Catalysts. Author manuscript; Available in PMC 2024 Aug 23. (PMC11343566; doi:10.3390/catal14010052)
Supplement: SI [file NIHMS1965123-supplement-SI.pdf]

catalysts

Supplementary Information

Engineering Electrode Polarity for Enhancing In Situ Generation of Hydroxyl Radicals Using Granular Activated Carbon

Stephanie Sarrouf [1], Amir Taqieddin [2], Muhammad Fahad Ehsan [1] and Akram N. Alshawabkeh [1,*]

[1] Department of Civil & Environmental Engineering, Northeastern University, Boston, MA 02115, USA; sarrouf.s@northeastern.edu (S.S.); m.ehsan@northeastern.edu (M.F.E.)
[2] Department of Mechanical & Industrial Engineering, Northeastern University, Boston, MA 02115, USA; taqieddin.a@northeastern.edu
* Correspondence: a.alshawabkeh@northeastern.edu

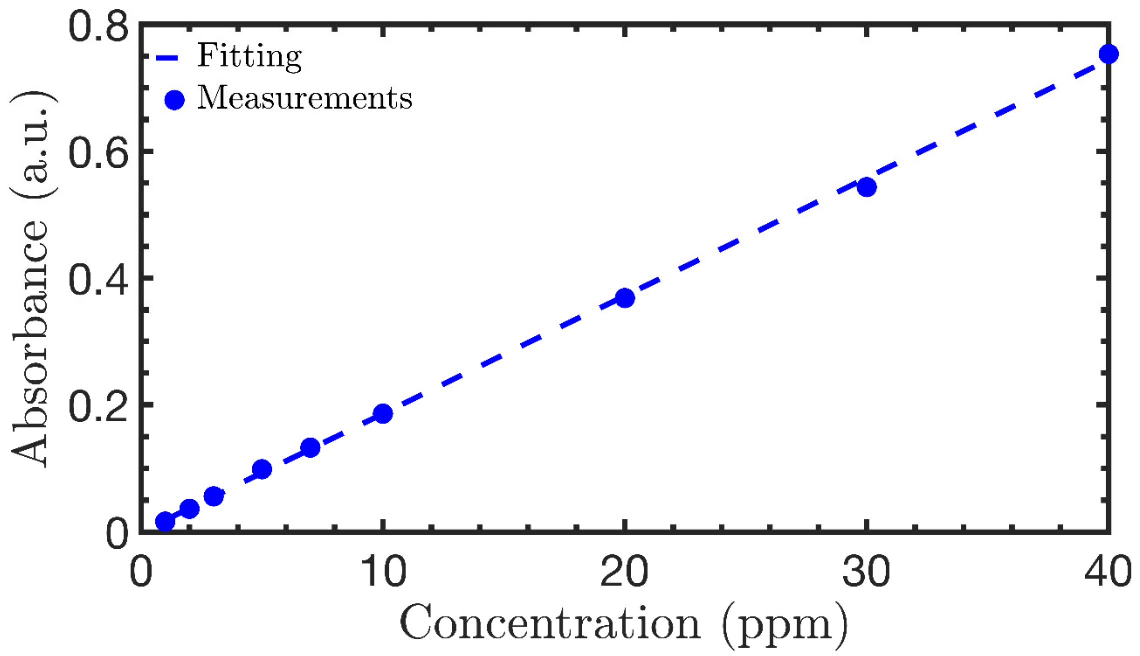

Figure S1. Calibration curve of H_2O_2 using spectrophotometer measured at a wavelength of 405 nm.

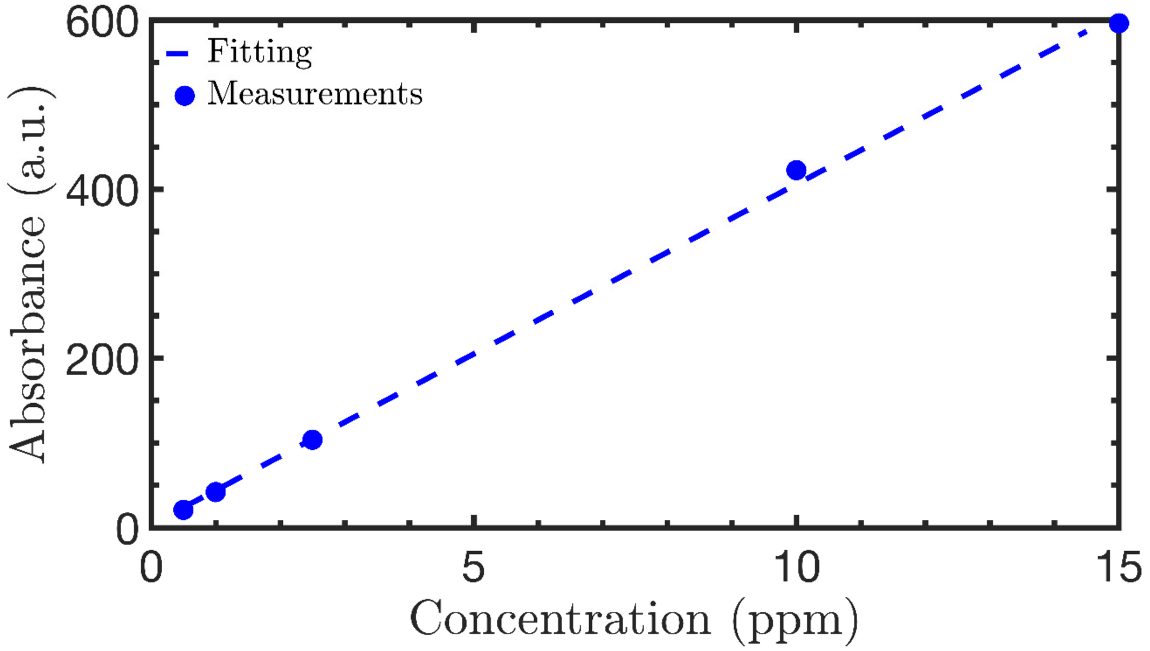

Figure S2. Calibration curve of 4-hydroxybenzoic acid used for the quantification of ·OH, using the HPLC.

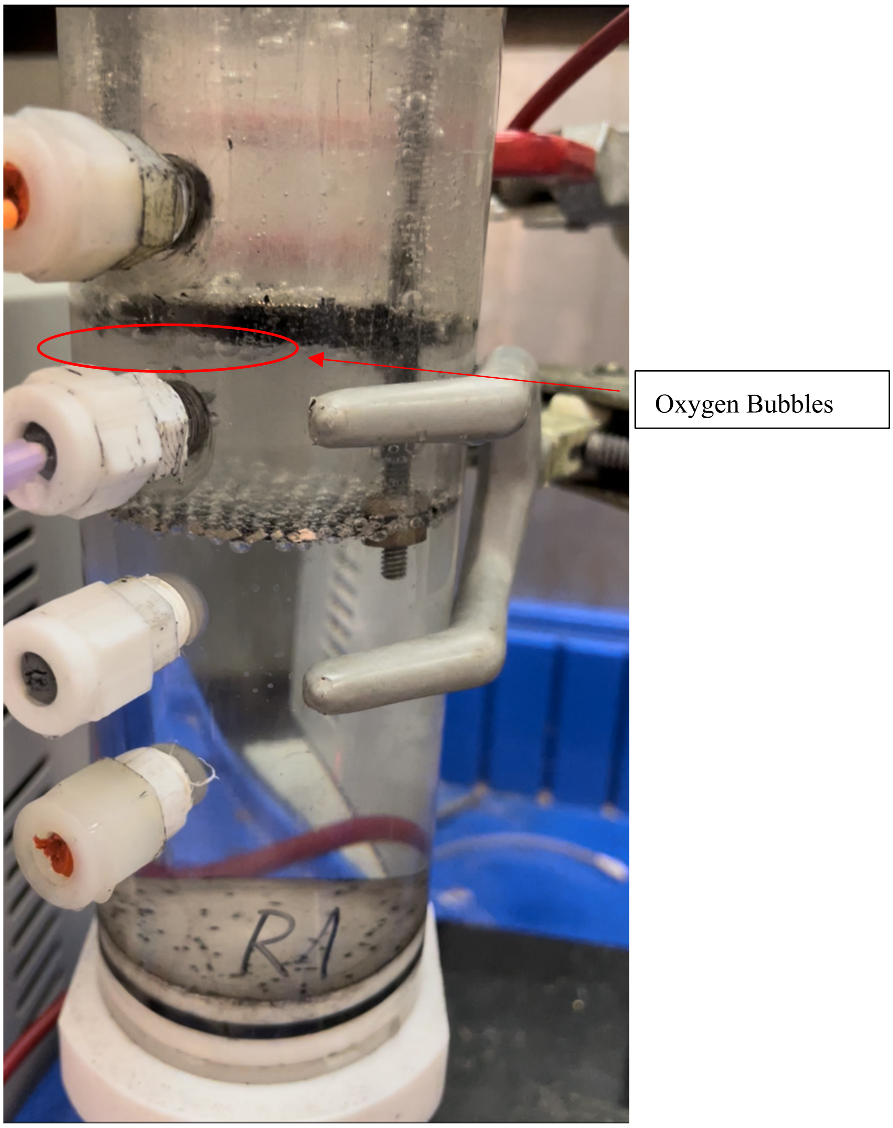

Figure S3. Electrochemical flow-through reactor showing the bubble accumulation at the surface of the cathode.
